# Knowledge and Attitude of the General Population About Do Not Resuscitate (DNR) in the Western Region, Saudi Arabia

**DOI:** 10.7759/cureus.44143

**Published:** 2023-08-26

**Authors:** Medhat Taha, Fatimah Obaid M Aldabali, Solaf Hilal Alotaibi, Rawya Zeed Melybari, Basel Abdulmonem Alqelaiti, Abdullah Mishal Alderhami, Taif Abdullah Bajaber

**Affiliations:** 1 Department of Anatomy, Al-Qunfudah Medical College, Umm Al-Qura University, Al-Qunfudah, SAU; 2 College of Medicine, Al-Qunfudah Medical College, Umm Al-Qura University, Al-Qunfudah, SAU; 3 College of Medicine, King Saud Bin Abdulaziz University for Health Sciences, Jeddah, SAU; 4 College of Medicine, Umm Al-Qura University, Makka, SAU; 5 College of Medicine, Taibah University, Madinah, SAU; 6 College of Medicine, Fakeeh College of Medical Sciences, Jeddah, SAU

**Keywords:** participants, western region, cardiopulmonary resuscitation, awareness, do not resuscitate

## Abstract

Background: A do-not-resuscitate (DNR) order is a medical order issued by a doctor. It directs medical professionals to refrain from performing cardiopulmonary resuscitation (CPR) if a patient's breathing or heartbeat ceases. Patients can refuse CPR in an emergency if they have a DNR order. The DNR order includes precise directives about CPR. Instructions for extra therapies like nourishment, other drugs, or painkillers are not included.

Aim: The aim of the study is to learn more about the western region's general population's knowledge and attitudes toward DNR orders and identify any challenges that may arise when dealing with DNR patients.

Methodology: A cross-sectional study was conducted in 2023 in the western region of Saudi Arabia. An online, self-administered questionnaire was distributed randomly from April 8, 2023 to June 6, 2023. The estimated sample size was 384, and 604 were the collected responses.

Results: A total of 383 (63.4%) participants were females, and 221 (36.6%) were males. Regarding the knowledge and attitude of the general population about DNR orders in the western region of Saudi Arabia, 276 (45.7%) study participants had satisfactory knowledge and awareness, while 328 (54.3%) had inadequate knowledge. A total of 343 (56.8%) participants thought that DNR is important; 255 (42.2%) felt that the DNR has reduced the pain of their relatives, and 181 (30%) believed that it has reduced the stress felt by the patient’s families. Of participants aged 20-30 years, 58.4% had satisfactory knowledge about DNR orders compared with those aged 50 and above; 76.1% of healthcare workers had satisfactory knowledge versus 26.5% of unemployed participants (*P*=.001).

Conclusion: We recommend increasing awareness and knowledge about DNR by conducting educational events about the concept and how to deal with patients who choose to acquire a DNR order.

## Introduction

When a patient experiences cardiac arrest, cardiopulmonary resuscitation (CPR), an emergency technique, is performed to save the patient's life [1]. Even when CPR resumes a patient's heartbeat or breathing, only a small percentage of patients fully recover. They might still require additional medical care, be critically ill, and never fully recover from their previous state of health. In addition, long-term damage to their brain or heart is possible. Do-not-resuscitate (DNR) forms are created for this reason. They indicate that patients will not receive any intervention that could prolong their lives or cause them to suffer [2]. A doctor issues a DNR order. If a patient's heartbeat or breathing stops, it instructs medical professionals not to perform CPR [3]. When it is anticipated that the patient will likely have a poor outcome, may not survive with CPR, or may survive with low function and quality of life after, medical professionals may occasionally recommend a DNR order [4]. A DNR is a predetermined order for patients to refuse CPR in an emergency. A DNR order includes precise directives about CPR. Instructions for other therapies like nourishment, drugs, or painkillers are not included. The patient, the proxy, or the patient's family are consulted before the doctor issues the DNR order [3]. The DNR decisions, based on various factors, including patient and relative preferences, ethical and legal issues, and the patient's state, have been the subject of numerous studies [5]. A study conducted in 2012 to assess the patient’s comprehension of DNR orders reported that of 429 patients, 84% had heard of the term DNR, and 56% thought the initial DNR discussions should occur when they were still in good health [6]. Moreover, according to an interventional study conducted in Switzerland, patients’ understanding of DNR was drastically enhanced after being provided information on new DNR orders' implementation and the DNR code of ethics [7]. Another study in Riyadh, Saudi Arabia, on 307 patients demonstrated that three-fourths of the participants were aware of the DNR order, 50% of whom correctly defined it. Additionally, 90% preferred discussing DNR when ill [8]. Further, a study evaluating the DNR order by interviewing 97 patients noted that 66% preferred sharing the decision with their family or doctor, and 58% had already discussed resuscitation with their physician [9]. The general population should be informed about DNR terminology. In this situation, the study aims to learn more about the general population's knowledge and attitudes toward DNR orders in the western region and identify any challenges that may arise when engaging with patients with a DNR order. 

## Materials and methods

Study design

A cross-sectional study was conducted in 2023 in Jeddah, Saudi Arabia. An online, self-administered questionnaire was distributed randomly from April 8 to June 6, 2023, to assess the general population's knowledge and attitude about a DNR order in the western region of Saudi Arabia.

Inclusion and exclusion criteria

The study included male and female subjects aged 18 and older who live in the western region of Saudi Arabia and excluded children under 18 years of age and those who are intellectually disabled.

Sample size and sampling procedure

The estimated population of the western region of Saudi Arabia is 11.27 million [10]. We used the public service of Creative Research Systems survey software to determine the precise population target of the sample size. This study's estimated sample size was 384 participants with a 95% confidence interval and a 5% margin of error; a p-value of less than 0.05 would be considered significant.

Questionnaire

An online, self-administered questionnaire was distributed randomly among people living in the western region of Saudi Arabia [4]. The questionnaire is divided into three sections: The first section contains consent and demographic information, and the second evaluates DNR knowledge. The third section assesses attitudes toward DNR orders.

Data collection and analysis

Data entry and statistical analysis were performed using IBM Corp. Released 2012. IBM SPSS Statistics for Windows, Version 21.0. Armonk, NY: IBM Corp. Descriptive statistics were applied in the form of tables and graphs, as appropriate. The chi-square test was used to highlight factors associated with study participants’ knowledge of DNR. Statistical significance was set at p<0.05.

Data analysis

The data were collected, reviewed, and fed into IBM Corp. Released 2012. IBM SPSS Statistics for Windows, Version 21.0. Armonk, NY: IBM Corp. All statistical methods used were two-tailed tests, with an alpha level of 0.05 considered significant if the p-value is less than or equal to 0.05. The overall level of knowledge regarding DNR orders was determined by summing up discrete scores for different correct and appropriate knowledge items. The overall knowledge score was categorized as unsatisfactory if the participants' score was less than 60% of the correct items and satisfactory if the overall score was 60% or more. Descriptive analysis was performed by prescribing frequency distributions and percentages for study variables, including participants' personal data, education, and employment. Also, knowledge regarding DNR orders was tabulated, while overall knowledge of their source of information was graphed. Cross tabulation to record factors associated with study participants' knowledge of DNR orders was conducted with a Pearson chi-square test for significance and an exact probability test if there were small frequency distributions.

## Results

A total of 604 participants eligible for this study completed the study questionnaire. Participants ranged from 18 to above 50 years old, with a mean age of 27.9 ± 12.7 years old. A total of 383 (63.4%) participants were female, and 588 (97.4%) were Saudi. As for employment, 276 (45.7%) were students, 214 (35.4%) were non-healthcare staff, and 46 (7.6%) were healthcare staff. There were 458 (75.8%) university graduates, while 118 (19.5%) had a secondary level of education, and 28 (4.6%) had a lower education level (Table 1).

**Table 1:** Personal data of study participants, Western region, Saudi Arabia

Personal data	No	%
Age in years		
< 20	71	11.8%
20-30	308	51.0%
31-40	69	11.4%
41-50	93	15.4%
> 50	63	10.4%
Gender		
Male	221	36.6%
Female	383	63.4%
Nationality		
Saudi	588	97.4%
Non-Saudi	16	2.6%
Employment		
Unemployed	68	11.3%
Student	276	45.7%
Non-health care staff	214	35.4%
Health care staff	46	7.6%
Educational level		
Below secondary	28	4.6%
Secondary	118	19.5%
University/above	458	75.8%

Of the study participants, 65.7% had heard of a DNR order. Also, 22.5% indicated that DNR is a medical order written by a doctor; it instructs healthcare providers not to perform CPR. Further, 12.6% of participants recorded that it is an order or prior instruction issued by a doctor or person authorized to avoid CPR, while 39.7% indicated “all” as the correct answer. Regarding the conditions where DNR is implemented, 59.4% opted for if the patient's condition is not fit for resuscitation, 42.5% chose if the patient's disease is incurable, not treatable, or death occurs, and 42.5% selected if resuscitation of the heart or lungs is not beneficial for a specific situation. Only 11.9% indicated that age is the only reason or justification for implementing a DNR order, while 46.9% chose that a DNR order affects other patients. A total of 52% of participants reported that the medical team is the best suited to decide about a DNR order, and 66.2% selected that the decision for a DNR order in a hospital or health facility should be taken if three consultants agree. Moreover, 59.4% of participants believed the patients had the right to decide on a DNR order if they gave prior consent, while 31% selected parents as decision-makers. As for the best time to make the decision about DNR, 41.1% selected in the case of brain death, 18.2% felt that it should be discussed before admission to the hospital or if the patient has a chronic disease, and 34.8% opted for the don’t know option (Table 2).

**Table 2:** Knowledge and awareness of the general population about ‘Do Not Resuscitate’ (DNR) in the Eastern Region, Saudi Arabia

Knowledge items		No	%
Have you ever heard of a Do Not Resuscitate (DNR)?	Yes	397	65.7%
	No	207	34.3%
What is Do Not Resuscitate (DNR)?	Medical order written by a doctor. It instructs healthcare providers not to do CPR	136	22.5%
	The order or prior instruction issued by a doctor or person authorized to avoid CPR	76	12.6%
	The patient himself asked not to try to resuscitate him	2	.3%
	Introducing the first and second, in addition to that it requires the consent of the patient	2	.3%
	All are correct	240	39.7%
	Others	13	2.2%
	Don't know	135	22.4%
What are the conditions where Do Not Resuscitate are done?	If the patient's condition is not fit for resuscitation	359	59.4%
	If the patient's disease is incurable, not treatable, death is achieved	257	42.5%
	If the patient is in a state of disability, or a state of mental inactivity, with chronic disease	68	11.3%
	If the patient had evidence of brain damage, difficult to treat	129	21.4%
	If resuscitation of the heart or the lung is not beneficial for a specific situation	257	42.5%
	Others	18	3.0%
	Don't know	126	20.9%
Age is the only reason or justification for applying for DNR.	Yes	72	11.9%
	No	394	65.2%
	Don't know	138	22.8%
Do you think DNR has a side effect on other patients?	Yes	283	46.9%
	No	139	23.0%
	Don't know	182	30.1%
Who is best decided for DNR?	Medical team	314	52.0%
	The patient (by prior decision) or one of his relatives	165	27.3%
	Another person	8	1.3%
	I don't think it should be taken at all	117	19.4%
Who has the right to decide on DNR in the hospital or any health facility?	When three consultants agree on the decision	400	66.2%
	The doctor supervising the patient's condition only	109	18.0%
	Don't know	95	15.7%
Who has the right to decide on DNR in the patient's family?	The patient himself, where gives prior consent to the decision	359	59.4%
	Parents	187	31.0%
	Brotherhood	8	1.3%
	Others	50	8.3%
When is the best time to decide on DNR?	In the case of brain death	248	41.1%
	It is discussed before admission to the hospital or if the patient has a chronic disease	110	18.2%
	If the patient's age is over 90	36	6.0%
	Don't know	210	34.8%

**Figure 1:**
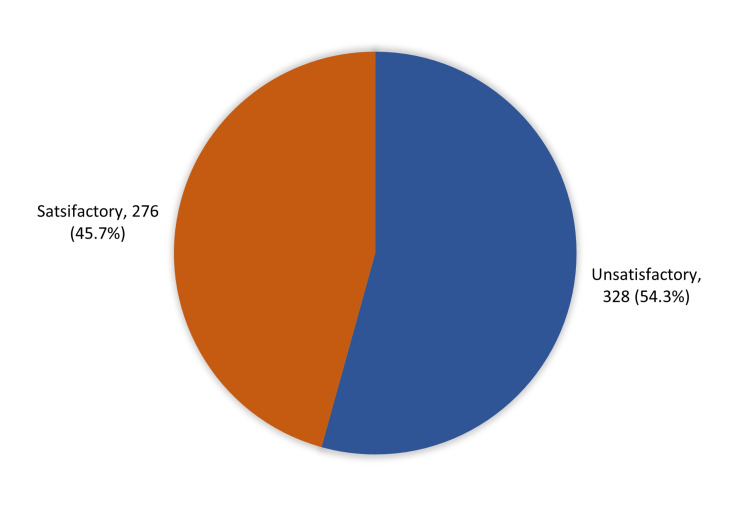
Overall, knowledge and awareness of the general population about ‘Do Not Resuscitate (DNR) in the Western Region of Saudi Arabia

Overall knowledge and awareness of the general population is shown in Figure 1. Of the respondents, 276 (45.7%) displayed satisfactory knowledge and awareness about DNR orders, while 328 (54.3%) had unsatisfactory knowledge.

Sources of information among study participants

**Figure 2:**
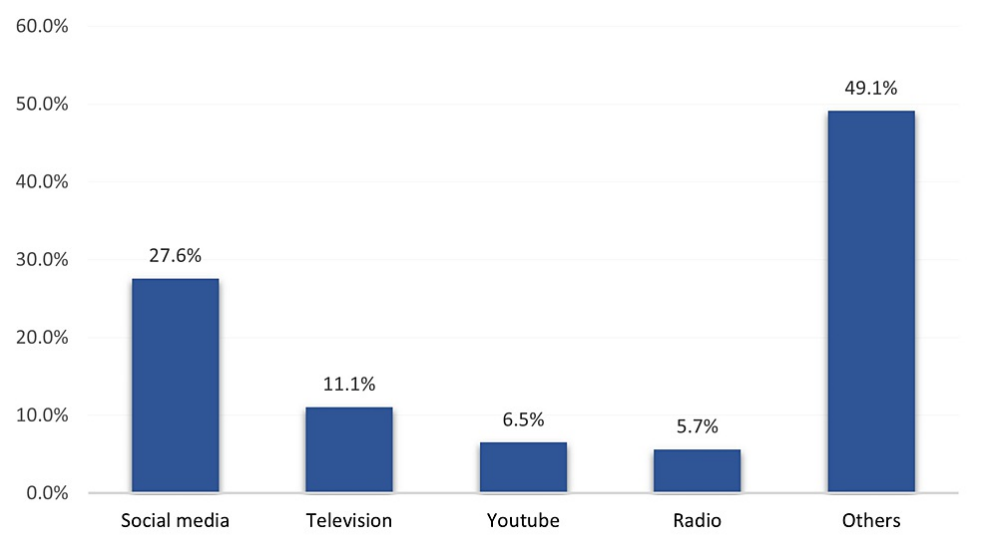
Source of information regarding Do Not Resuscitate (DNR) among study participants

The most reported sources of information were social media (27.6%), TV (11.1%), YouTube (6.5%), and radio (5.7%) (Figure 2).

A total of 56.8% of participants thought that DNR orders were important: 42.2% felt that DNR orders reduced pain, and 30% believed they would reduce the stress faced by the patient’s family (Table 3).

**Table 3:** The attitude of the general population about Do Not Resuscitate (DNR) in the Western Region, Saudi Arabia

Attitude	No	%
Do you think that "Do Not Resuscitate (DNR)" is important?		
Yes	343	56.8%
No	91	15.1%
Don't know	170	28.1%
Do you feel that DNR reduces pain?		
Yes	255	42.2%
No	145	24.0%
Don't know	204	33.8%
If DNR is implemented in a patient, will this reduce the stresses facing his family?		
Yes	181	30.0%
No	206	34.1%
Don't know	217	35.9%

Precisely 20.7% of participants indicated that they would agree to a DNR order for one of their relatives if necessary, and 26.3% might agree. Only 10.4% of the study participants reported having a DNR order for a relative before 12 years, among 41.3%, and before five years, among 33.3% of them. Furthermore, 38 (60.3%) participants reported feeling sad after the decision, and 10 (15.9%) were relieved because of the relief to the patient (Table 4).

**Table 4:** Personal experience and practice of DNR among study participants, Western region, Saudi Arabia

Practice	No	%
Will you agree to the decision of DNR for one of your relatives, if necessary?		
Yes	125	20.7%
Maybe	159	26.3%
No	320	53.0%
Have you ever had a DNR decision for a relative?		
Yes	63	10.4%
No	467	77.3%
Don't know	74	12.3%
If yes, when was that? (n=63)		
Before 1 year	26	41.3%
Before 5 years	21	33.3%
Before 10 years	9	14.3%
More than 10 years	7	11.1%
What was your feeling? (n=63)		
Felt relieved for him	10	15.9%
Felt sadness	38	60.3%
Was surprised	4	6.3%
Others	11	17.5%

Of the participants aged 20-30, 58.4% had a satisfactory knowledge level about DNR orders, compared to 28.6% for those above 50 years with recorded statistical significance (P=.001). Also, 76.1% of healthcare workers had satisfactory knowledge versus 26.5% of unemployed participants (p =.001). Satisfactory knowledge about DNR was detected among 68.6% of participants who received their information from TV, compared to 38.5% of others who selected radio (p=.009). Likewise, 54% of participants who had made DNR decisions for relatives had satisfactory knowledge about the procedure compared to 47.3% of those who did not (p=.004) (Table 5).

**Table 5:** Factors associated with participants' knowledge about Do Not Resuscitate (DNR) P: Pearson X2 test, $: Exact probability test, * P < 0.05 (significant)

Factors	Overall awareness and perception level				p-value
	Unsatisfactory		Satisfactory		
	No	%	No	%	
Age in years					.001*
< 20	45	63.4%	26	36.6%	
20-30	128	41.6%	180	58.4%	
31-40	49	71.0%	20	29.0%	
41-50	61	65.6%	32	34.4%	
> 50	45	71.4%	18	28.6%	
Gender					.998
Male	120	54.3%	101	45.7%	
Female	208	54.3%	175	45.7%	
Employment					.001*
Unemployed	50	73.5%	18	26.5%	
Student	128	46.4%	148	53.6%	
Non-health care staff	139	65.0%	75	35.0%	
Health care staff	11	23.9%	35	76.1%	
Educational level					.427
Below secondary	18	64.3%	10	35.7%	
Secondary	67	56.8%	51	43.2%	
University/above	243	53.1%	215	46.9%	
Source of information about DNR					.009*$
Television	16	31.4%	35	68.6%	
Radio	16	61.5%	10	38.5%	
Social media	47	37.0%	80	63.0%	
YouTube	11	36.7%	19	63.3%	
Others	114	50.4%	112	49.6%	
Have you ever had a DNR decision for a relative?					.004*
Yes	29	46.0%	34	54.0%	
No	246	52.7%	221	47.3%	
Don't know	53	71.6%	21	28.4%	

## Discussion

In this study, we attempted to evaluate awareness, knowledge, and attitudes about DNR orders and identify challenges that may arise when treating patients with a DNR order in place. To our knowledge, no previous study in this region has reviewed a similar subject.

‏There were 604 participants in the study. The results indicate that most participants had never heard of DNR (65.7%) and could not identify the correct definition (39.7%). ‏In a previous Saudi study report from Jeddah, it was discovered that there was poor knowledge and understanding of resuscitation [11]. This agreed with the study's findings. 

‏The sources of information reported by the study participants indicated that 27.6% received their information from social media applications, considered the least reliable sources of information. ‏A study in Jeddah reported that 34.3% of participants had heard the term DNR on social media [12], supporting our results. Most participants strongly agreed that patients should be included in decision-making, whereas 31% chose parents as decision-makers. According to a study in Hong Kong, most participants believed that the patient's preferences should come first when making a DNR choice, followed by the family's wishes and then the patient's social status [13]. When asked to define DNR correctly, approximately 22.5% of the participants selected "Medical order written by a doctor. It instructs healthcare providers not to do CPR." Research from Riyadh, Saudi Arabia, supports our findings, revealing that 44.0% of participants had a similar opinion about a DNR order [14]. In this study, regarding conditions where a DNR order was obtained, we discovered that more than half of our participants selected the option indicating that a patient’s condition was not suited for resuscitation. Compared to the research in the Aseer region, they observed that 42.58% of their participants chose the same reason. Also, we discovered that 42.5% of our participants chose the following conditions to consider a DNR order: if a patient’s disease is incurable, not treatable, or death occurs; in the Aseer region study, 6.69% of their participants opted for the same reason [4].

In our research, we discovered that 56.8% of the participants believed a DNR order was important; this was corroborated by a study in the Aseer region of Saudi Arabia, which concluded that more than half of the participants believed a DNR order was important. However, we observed that less than half of our participants believed that DNR reduces pain; in contrast, more than half of the participants in the Aseer region study agreed with it. Lastly, fewer than half of our participants believed that implementing a DNR order would ease the strain on the patient’s family; in contrast, the Aseer region study had more than half of their participants believe that [4]. The study illustrated that 53% of the participants didn’t consider a DNR order as an option for their relatives, and only 20.7% were willing to agree. Only 10.7% (63 of the 604 participants) had a DNR order in place for a relative; 74.6% had procured a DNR order for a relative in the past five years, and most participants (60.3%) felt sad about that decision. Some participants (15.9%) felt relieved for their relatives by ending their suffering. The study also highlighted that most of our participants belonged to the age group of 20-30 years; it revealed that 58.4% of the participants, which is the majority, had good awareness levels of DNR. It also showed that the health workers are more familiar with, understand more, and are better educated about DNR orders by 76.1% compared to the unemployed. Mainly satisfactory knowledge came from TV (68.6%), in contrast to the information from the radio, where only 38.5% of them were satisfied. Additionally, most of the participants with a DNR order for a relative were able to define and understand DNR better by 54% than those who didn’t. And in general, despite the difference between the sample size in our study and the study in Vancouver, the results on average and percentage differ vastly; the percentage of satisfying knowledge also has a huge variation [6].

Limitations

Limited validation was conducted on our survey instrument. It is possible that a large percentage of respondents misidentified the term DNR due to the way the question was worded rather than a lack of knowledge. It was a small study; thus, the findings could not be generalized to populations in suburban, rural, and other areas. Religion and education weren't investigated in this study, which may be significant factors in patients' attitudes regarding this topic.

## Conclusions

Of the people living in the western region of Saudi Arabia, 45.7% have good knowledge and awareness about DNR orders, while 45.3% have poor knowledge and awareness. We recommend increasing awareness and knowledge about DNR orders by conducting educational events about the approach to DNR and how to engage with patients who opt for a DNR order.
